# Genetic differences between wild and hatchery‐bred brown trout (*Salmo trutta* L.) in single nucleotide polymorphisms linked to selective traits

**DOI:** 10.1002/ece3.3070

**Published:** 2017-05-30

**Authors:** Arne N. Linløkken, Thrond O. Haugen, Matthew P. Kent, Sigbjørn Lien

**Affiliations:** ^1^ Faculty of Education and Natural SciencesInland Norway University of Applied SciencesHamarNorway; ^2^ Faculty of Environmental Sciences and Natural Resource ManagementNorwegian University of Life SciencesÅsNorway; ^3^ Department of Animal and Aquacultural SciencesNorwegian University of Life SciencesÅsNorway

**Keywords:** body size, natural selection, outlier *F*_ST_, salmonid, SNPs

## Abstract

To study effects from natural selection acting on brown trout in a natural stream habitat compared with a hatchery environment, 3,781 single nucleotide polymorphism (SNP) markers were analyzed in three closely related groups of brown trout (*Salmo trutta* L.). Autumn (W/0+, *n *=* *48) and consecutive spring (W/1+, *n *=* *47) samples of brown trout individuals belonging to the same cohort and stream were retrieved using electrofishing. A third group (H/1+, *n *=* *48) comprised hatchery‐reared individuals, bred from a mixture of wild parents of the strain of the two former groups and from a neighboring stream. Pairwise analysis of *F*_ST_ outliers and analysis under a hierarchical model by means of ARLEQUIN software detected 421 (10.8%) candidates of selection, before multitest correction. BAYESCAN software detected 10 candidate loci, all of which were included among the ARLEQUIN candidate loci. Body length was significantly different across genotypes at 10 candidate loci in the W/0+, at 34 candidate loci in the W/1+ and at 21 candidate loci in the H/1+ group. The W/1+ sample was tested for genotype‐specific body length at all loci, and significant differences were found in 10.6% of all loci, and of these, 14.2% had higher frequency of the largest genotype in the W/1+ sample than in W/0+. The corresponding proportion among the candidate loci of W/1+ was 22.7% with genotype‐specific body length, and 88.2% of these had increased frequency of the largest genotype from W/0+ to W/1+, indicating a linkage between these loci and traits affecting growth and survival under this stream's environmental conditions. Bayesian structuring of all loci, and of the noncandidate loci suggested two (*K *=* *2), alternatively four clusters (*K *=* *4). This differed from the candidate SNPs, which suggested only two clusters. In both cases, the hatchery fish dominated one cluster, and body length of W/1+ fish was positively correlated with membership of one cluster both from the *K *=* *2 and the *K *=* *4 structure. Our analysis demonstrates profound genetic differentiation that can be linked to differential selection on a fitness‐related trait (individual growth) in brown trout living under natural vs. hatchery conditions. Candidate SNP loci linked to genes affecting individual growth were identified and provide important inputs into future mapping of the genetic basis of brown trout body size selection.

## 1 INTRODUCTION

In captive breeding of brown trout (*Salmo trutta* L.) for conservation, supplemental stocking or farming, wild specimens are captured, gametes striped, and fertilization conducted artificially. For conservation and supplemental stocking, each generation is most often bred from wild parents as captive breeding affects the gene pool and the individual fitness expressed as reduced survival and recruitment, potentially caused by the lack of selective forces in captivity due to high survival compared to fish in a natural environment (Araki, Berejikian, Ford, & Blouin, 2008; Araki, Cooper, & Blouin, 2007; Saikkonen, Kekalainen, & Piironen, 2011).

A variety of assay tools, analysis techniques and software packages are available for geneticists studying topics related to conservation biology and molecular ecology, with both simple sequence repeats (SSRs) (Balloux & Lugon‐Moulin, 2002) and single nucleotide polymorphisms (SNPs) (Thomas & Kejariwal, 2004) representing powerful tools for genetic studies. SNPs are prevalently biallelic in contrast to SSRs; however, SNP assays are easy to standardize across detection platforms and laboratories and may be developed so that thousands of robust markers are genotyped simultaneously in a single sample. Furthermore, while SSR loci are typically selectively neutral, the wide availability of SNPs implies that a study may include loci affected by selection, thereby providing additional functional information pertinent to adaptation (Brooks et al., 2010; Davoli et al., 2003; Kolbehdari et al., 2008). Brown trout are present in streams and lakes of different environmental conditions and are adapted to local environments through phenotypic plasticity (Valiente, Juanes, Nuñez, & Garcia‐Vazquez, 2010), and genetic modification due to natural selection (Jensen et al., 2008). An important trait of animals is individual growth (Stearns, 1992), and being indefinite in fish, growth shows high variability due to the ultimate environmental factors, among which temperature is crucial (Bærum, Vøllestad, Kiffney, Rémy, & Haugen, 2016; Jensen, Forseth, & Johnsen, 2000; Jensen et al., 2008; Nicola & Almodovar, 2004). In monitoring populations from a conservation perspective, important population‐genetic indices are calculated based on SSRs or SNPs, but to explore the effects of selection, SNPs are better suited than the basically neutral SSRs.

**Figure 1:**
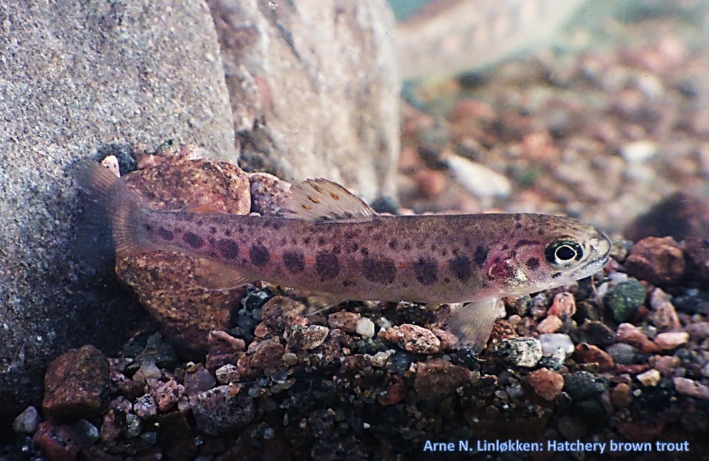
One‐year old hatchery brown trout (*Salmo trutta* L.) in aquarium

This study includes three groups of brown trout, from the same population, of which two groups comprise wild specimens and one is composed of F1‐generation individuals reared in a hatchery. The two wild fish groups were sampled in order to study effects of over‐winter size‐selective survival (selective sweeps) among loci of SNP markers. The hatchery‐reared fish (Figure 1) are used for annual supportive stocking in a downstream lake and are bred from a mixture of two local strains to maintain locally adapted genotypes. One of those is the wild fish strain of the two former groups. The hatchery group was included to explore the differing effects of selective forces in wild compared with hatchery fish bred from a limited number of randomly picked wild fish subject to forced mating, and with offspring living in a protected environment. Body size, which is shown to correlate positively with survival of young fish (Lorenzen, 1996), is used as a selective trait in the comparisons.

The relationship between body length and genotypes is studied, highlighting the differences between brown trout of the same population and cohort caught at different ages, and the differences between wild and hatchery‐reared fish of the same cohort. Population‐genetic analysis and assignment to clusters were performed, and evidence of bottleneck events was explored in order to characterize populations. The following hypothesis were tested as follows: (1) Genetic differentiation between different age groups of the same cohort and population, is in part affected by selective forces, potentially linked to selective traits such as body size, that is, individual growth. (2) Artificial spawning and breeding in hatchery will, due to the lack of sexual selection and natural selection by the environment, result in a “hatchery genepool” differing from that of their pristine relatives.

## 2 METHODS

### 2.1 Study area and sampling

The study is based on genotyping data obtained from brown trout belonging to one of three sample groups comprising 48 specimens from the same tributary to the Lake Savalen. The first two groups consisted of wild first‐year (W/0+) and 1‐year old (W/1+) brown trout from the same 2011 cohort and population. They were sampled by means of electro fishing (a portable apparatus powered by a 12 V battery) in the same stretch of Sagbekken (EPSG 4326: 62.319°N; 10.486°E), a small tributary (conductivity 4.0 mS/m) of Lake Savalen in central Norway. The sampling was conducted September 29, 2011, at water temperature 6°C, and June 15, 2012, at water temperature 11°C. The water discharge was approximately similar at the two sampling occasions, so catchability was potentially lower for the W/0+ group due to lower temperature and smaller sized fish (Bohlin, Hamrin, Heggberget, Rasmussen, & Saltveit, 1989) compared with the W/1+ sampling. The third group consisted of 1‐year‐old hatchery fish (H/1+) sampled from Evenstad hatchery (EPSG 4326: 62.424°N; 11.1005°E) as randomly as possible from the breeding tank by means of a landing net. Effective number of breeders *N*_b_ of the sample groups W/0+, W/1+, and H/1+ have been estimated to be 38, 35, and 18, respectively, based on SSR and linkage disequilibrium, and the number of full‐sibs pairs was 45%–180% higher in the H/1+ than in the wild fish samples (Linløkken, Haugen, Mathew, Johansen, & Lien, 2016). Fish length was measured (mm, from the snout to the tip of the tail fin in natural position) as the only detectable trait in these samples. In June 2012, scales were sampled from specimens >90 mm to ensure age, and one specimen was suspected to be 2+ years of age and therefore excluded (47 samples of W/1+ specimens remained).

A total of 24 wild brood parents (11 females and 13 males) provided gametes that were randomly mixed to produce the H/1+ offspring. The brood parents were collected from two streams, Sagbekken and Mogardsbekken, whose confluence is 800 m downstream from the wild fish sampling site in Sagbekken and flows into lake Savalen 900 m downstream (EPSG 4326: 62.312°N; 10.505°E) of the confluence. The survival rate of the hatchery group was >95% from hatching to sampling. SSR‐based analysis of eight loci has shown low, but significant neutral genetic differentiation (*F*_ST_ **=* *0.013, 95% *C.L. *=* *0.003–0.0023) between brown trout from Sagbekken and Mogardsbekken (Linløkken & Johansen, 2010).

### 2.2 DNA extraction and isolation

Genomic DNA was extracted from caudal fin clips and preserved in 96% EtOH at −20°C, using a Blood & Tissue Kit (Qiagen, Hilden, Germany). From 30 μl cleared lysate, total genomic DNA was isolated using GenoM‐48 Robotic Workstation (GenoVision, Oslo, Norway) and magnetic bead technology. Binding of DNA to magnetic beads (Qiagen) was performed in 200 μl buffer MDL (MagAttract DNA Blood M96 kit; Qiagen) after which beads were washed twice in 200 μl of 80% EtOH, GenoPrep wash solution (GenoVision, Toyobo Kita‐ku, Osaka, Japan) and water, before finally eluting DNA in 0.1× TE buffer at pH 8.0. The purity and concentration of the DNA samples were determined spectrophotometrically using a NanoDrop ND‐1000 (NanoDrop Technologies, Wilmington, DE, USA).

### 2.3 SNPs

Single nucleotide polymorphism genotyping was performed according to manufacturer's instructions using an Illumina iSelect SNP‐array containing 5,509 SNP assays. Briefly, this array included SNPs detected in whole‐genome sequencing data obtained from 16 individuals representing both domestic families and wild populations. Extensive filtering was performed before choosing a final set of markers. This began by identifying 47,000 SNPs who shared the following characteristics, (1) a minimum of 2 reads representing the minor allele in at least two individuals, (2) one homozygous individual with a minimum of four reads, (3) a minimum of 60 bp to the closest SNP or indel, (4) no A/T or C/G variants, and (5) biallelic. A subset of this selection was included on the array; 56% of the content includes SNPs distributed evenly across de novo sequence contigs >7,750 bp, 21% are *S.trutta* SNPs mapping to *S.salar* full length cDNA sequences, 14% are SNPs within *S.trutta* contigs sharing high sequence similarity with *S.salar* contigs (Lien et al., 2011), 5% are *S.salar* SNP assays known to function on *S. trutta* DNA, 2% were SNP pairs from smaller *S.trutta* contigs (<11 kb), the remaining SNPs were chosen from contigs with similarity to specific candidate genes. The majority of SNPs were assigned to one of the 40 linkage groups (LGs) expected in this species (2*n *=* *80) (Phillips & Rab, 2001), except 221 SNPs, which are so far unassigned (Table S1, S. Lien, unpublished).

Using a larger set of reference samples, markers were manually inspected using GenomeStudio (version 2011.1, Illumina Inc., San Diego, CA, USA) and classified as “SNP,” multisite‐variant (“MSV3”), or “other” based on their cluster patterns. A SNP was defined as presenting three genotype clusters (AA, AB, BB) with theta positions at 0.0, 0.5, and 1.0, that is, a typical single locus, diploid marker. A marker was classified as MSV3 when it showed the same three clusters but that these were skewed so that theta positions are 0.0, 0.25, 0.5, or 0.5, 0.75, 1.0, that is, a duplicated locus marker where alleles are fixed at one position. “Other” included markers with low polymorphism rates, failed genotyping assays. The average genotyping call rate on a per sample basis was 99.48%, with a range from 93.41% to 99.81%, and no samples were excluded from the analysis.

### 2.4 Statistical analysis

Data files were transformed to appropriate formats by means of the PGDSpider (version 2.1.1.0) software (Lischer & Excoffier, 2012), and the detection of candidate markers under selection was performed by means of two different softwares, ARLEQUIN 3.5.1.2 (Excoffier & Lischer, 2010) and BAYESCAN (Foll & Gaggiotti, 2008). The latter is shown to produce lower error rates in simulated datasets (Narum & Hess, 2011), and lower number of outliers in empirical datasets than the ARLEQUIN method (Tsumura et al., 2014), that is, it performs a more conservative statistic. Both methods are based on locus‐specific genetic differentiation (*F*_ST_) outliers to detect candidate markers under selection (Beaumont & Nichols, 1996) and were both used to analyze the sample groups pairwise, and in one group. The ARLEQUIN was also used to perform analyzes with a hierarchic simulation model, by grouping wild specimens (W/0+ and W/1+) compared with the hatchery group (H/1+), analyzed with a hierarchical simulation model. The hierarchic model is supposed to be the most suited for populations sharing recent common ancestry, reducing the number of false‐positive outlier loci (Excoffier, Hofer, & Foll, 2009). In all cases, the default 100 simulated demes and 20,000 coalescent simulations were used.

Global and pairwise genetic differentiations (*F*_ST_) were estimated by means of the ARLEQUIN software, and the pairwise differentiation was calculated for all SNP loci, and separately for SNP loci detected as candidates under selection and the loci that were not detected, to explore effects of selection on genetic structuring. To explore the false discovery rates (FDR), all *p*‐values from ARLEQUIN of nonmonomorphic loci were put into the computer program SGoF+ to correct test values (Carvajal‐Rodriguez & de Uña‐Alvarez, 2011). The previous version of this software, SGoF (included in the new version), calculates a multiple hypothesis testing adjustment using a sequential goodness of fit metatest, that is, especially designed for molecular biology applications where large numbers of tests are performed (Carvajal‐Rodríguez, de Uña‐Alvarez, & Rolán‐Alvarez, 2009). SGoF+ uses the maximum distance between a uniform distribution of *p‐*values, and the observed distribution resulting in an improvement in the statistical power to reject the null hypothesis when it is false, that is, it performs a less conservative statistic than SGoF. The software also estimates the *q* value (FDR) for each test (Carvajal‐Rodriguez & de Uña‐Alvarez, 2011). Candidate loci of selection detected by means of the BAYESCAN software (Foll & Gaggiotti, 2008), and the Bayes factor (BF), that is, the relationship between models of selection and neutrality based on Jeffreys’ scale of evidence for BF. The log10(BF) was used as criteria, and according to Jeffreys’ interpretation, log10(BF)* *=* *0.5–1.0, 1.0–5, 1.5–2.0, and >2.0 are characterized, respectively, as substantial, strong, very strong, and decisive evidence for selection (Foll & Gaggiotti, 2008).

The software STRUCTURE 2.3.4 (Pritchard, Stephens, & Donnelly, 2000) was used to infer the most likely number of population clusters (*K*) constituting each sample. Each individual *i* was assigned a membership coefficient (*Q*_i_) for each inferred cluster and was assigned to the cluster of highest *Q*; and each sample group was given a proportion of membership in each cluster. The analysis was performed for all SNPs with outlier *F*_ST,_ and those with *F*_ST_ within 95% confidence limits were analyzed separately to explore the potential effects of selection on the genetic structure.Ten independent runs were performed for each *K* (1–7) simulated, assuming an admixture model and correlated allele frequency. The admixture model assumes that individuals have inherited fractions of their genome from more than one population and is recommended as a starting point by Pritchard et al. (2000), whereas the correlated allele frequency model is a default. A burn‐in period of 50,000 iterations and a Monte Carlo Markov Chain (MCMC) of 50,000 iterations were used. The most likely number of clusters *K* in all simulations was assumed to be in the range of *K *=* *1 to *K *= *n* + 3 (where *n* is the number of populations sampled), as described by Evanno, Regnaut, and Goudet (2005), attained by means of Structure Harvester (Earl & vonHoldt, 2012) (available at http://taylor0.biology.ucla.edu/structureHarvester/). The estimated cluster membership coefficient matrices, for individuals and sampling groups, for the best fitted *K* was permuted so that all replicates have as close a match as possible using the CLUMPP program (Jakobsson & Rosenberg, 2007). Linear models were fitted to body length and individual cluster membership (*Q*_i_) of fish within each group W/0+, W/1+, and H/1+ using the R software (R Core Team, 2012). The W/1+ sample, having survived the first year in its natural stream habitat, was of special interest regarding body size/growth and genotype, and all nonmonomorphic loci were, therefore, tested for length differences between genotypes. One‐way ANOVA was conducted with length as the response variable, and locus (with levels *AA*,* AG,* and *GG* or *AA*,* AC,* and *CC*) as predictor, and Tukey pairwise post hoc test was conducted, testing mean body length across genotypes of the loci. This test was also conducted in the W/0+ and H/1+ groups on loci with significant genotypic lengths in the W/1+ group and in loci detected as candidates of selection. The distribution of genotypes at loci with significant length differences between two genotypes was compared between W/0+ and W/1+ and tested with Fisher exact test by means of the R software (R Core Team, 2012), when at least three test groups (genotype within sample) were represented by ≥5 individuals. The r package VennDiagram (Chen, 2016) was used to illustrate number of loci detected in the ARLEQUIN *F*_ST_ outlier analysis.

The BOTTLENECK 1.2.02 software (Cornuet & Luikart, 1996) was run using an infinite allele mutation model (I.A.M.), a stepwise mutation model (S.M.M.), and a two‐phase mutation model (T.P.M.). Populations exhibiting a significant number of loci with heterozygote excess by means of a Wilcoxon sign‐rank test have likely undergone a recent population bottleneck event.

## 3 RESULTS

Totally, 3,871 SNP loci were analyzed (Table S1) with scoring success of 97.6 to 99.4% within each sample, and 3,196 (H/1+) to 3,270 (W/1+) of 3,779 to 3,861 successfully analyzed SNP loci were polymorphic, including 7,067 to 7,141 alleles. The mean body length (±SD) of the sample groups W/0+, W/1+, and H/1+ was 51.3 (±4.9) mm, 70.7 (±15.3) mm, and 101.4 (±9.9) mm, respectively, with coefficients of variation 9.6, 18.5, and 9.8, respectively. H/1+ on average 30.7 mm larger than W/1+, W/1+ was on average 19.4 mm larger than W/0+, and W/1+ had the largest coefficient of variation. The variance of the means was different (Levene's test for homogeneity of variance, *F*_2,140_ **=* *12.27, *p *<* *.0001), and one‐way ANOVA of means (not assuming equal variances) was performed, revealing significant differences between the sample groups (Welsh ANOVA: *F*_2,79.5_ **=* *497.58, *p *<* *.0001), and Tukey post hoc test stated significant differences between all pairs (*p *<* *.01).

### 3.1 Body size and genetic selection

Of the totally 3,270 bi‐allelic loci of the W/1+ sample (Tables 1 and S2–S4), ANOVA and Tukey post hoc tests revealed significant mean length differences between at least two genotypes in 345 (10.6%) loci, when omitting test groups including <5 specimens of a genotype. In 49 (14.2%) of these loci, the largest genotype of W/1+ was significantly more frequent in the W/1+ sample than in the W/0+ (Table 1).

**Table 1:** Number of loci (*S*) with genotypic length differences in the W/1+ sample (*S*_∆L_) and loci with higher genotype frequency (Fisher's exact test *p* < .05) of the largest genotype in W/1+ compared with the frequency in W/0+ (*F*_*p* < .05_) of locus in percent of group of loci

Groups of loci	*S*	*S*_∆L_	%	*F*_*p* < .05_	%
All biallelic loci of W/1+	3270	345	10.6	49	14.2
Candidates of pairwise W/0+ vs. W/1+ analysis	150	34	22.7	30	88.2
Candidates of pairwise W/1+ vs. H/1+ analysis	199	24	12.1	3	12.5
Candidates of pairwise W/1+ vs. H/1+ excluding ∩(W/0+ vs. W/1+)	184	21	11.4	1	4.8
Candidates of pairwise W/1+ vs. H/1+ SGoF corrected	19	4	21.1	0	0
Candidates of hierarchic model	203	18	8.9	2	11.1
Candidates of hierarchic model SGoF corrected	26	4	15.4	1	25.0

**Figure 2:**
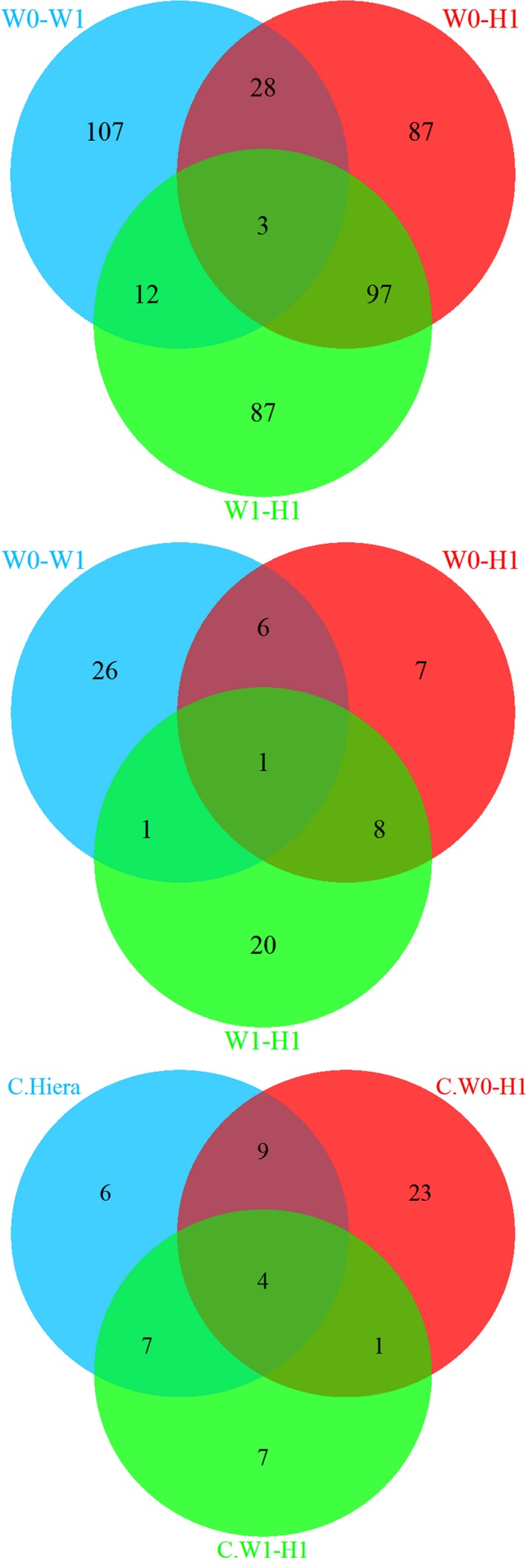
Venn diagram representing the number of SNPs detected as candidates of selection by pairwise analysis of three sets of samples (W/0+ vs. W/1+, W/0+ vs. H/1+, and W/1+ vs. H/1+) (upper panel), and the number of these candidate loci where significant difference between mean body length of at least two genotypes was revealed (central panel). The number of candidate loci of three sets, including the hierarchic model analysis, after SGoF correction (lower panel)

**Figure 3:**
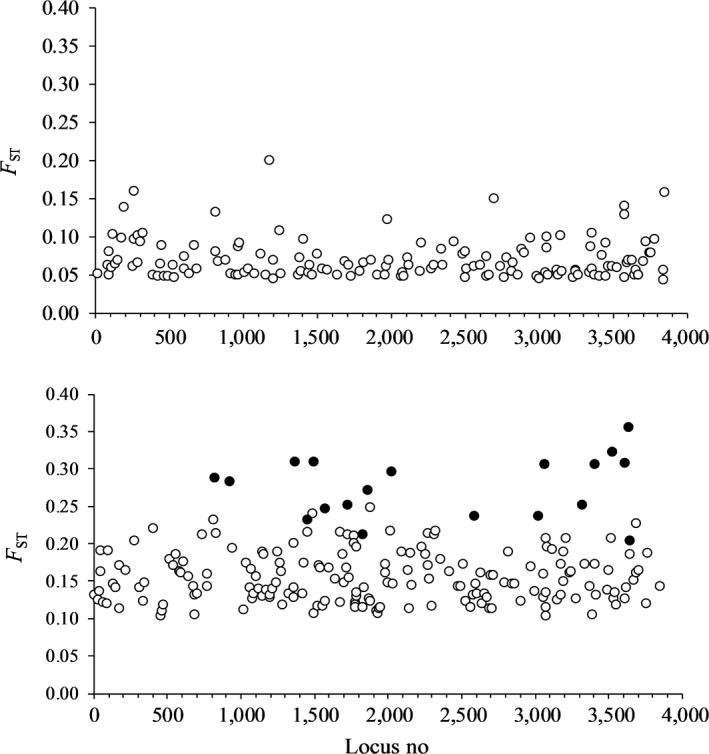
Significant outlier *F*_ST_ in pairwise ARLQUIN (○) analysis for the W/0+ vs. W/1+ pair (upper panel) and for the W/1+ vs. H/1+ pair (lower panel, ● = significant after SGoF correction) plotted against locus number

Pairwise ARLEQUIN analyzes detected 150 to 215 loci (4.5–7.0% of the pairs of bi‐allelic loci) as candidates of positive selection by significant outlier *F*_ST_ (*p *<* *.05), before correction (Table S2). *F*_ST_ was lowest for the W/0+ vs. W/1+ pair, and highest for W/0+ vs. H/1+ (Figure 2), and whereas most of the significant outliers of the W/0+ vs. W/1+ ranged 0.05–0.10, those from the W/0+ vs. H/1+ set ranged 0.10–0.25. Most of the candidate loci, still significant after SGoF correction, had *F*_ST_ > 0.20 (Figure 3). The number of exclusive candidate loci was highest in the W/0+ vs. W/1+ set (107), and the number of loci overlapping between the sets was highest between the two sets involving the H/1+ sample (112 and 128 loci). All *F*_ST_ outliers with *p *<* *.05 in the ARLEQUIN analysis suggested positive selection, and all were significant after SGoF+ correction. With the more conservative SGoF correction, no test was significant for the W/0+ vs. W/1+ set (Table S2). In the W/0+ vs. H/1+ set, 215 (7%) SNPs were detected candidates of positive selection, and 37 (1.2%) was significant after SGoF correction (Figure 2). A total of 199 (6.4%) candidates of positive selection were detected in the W/1+ vs. H/1+ set, and 19 (0.6%) outliers were significant after SGoF correction.

Among the 150 candidate loci detected in the W/0+ vs. W/1+ set, 34 (20%, or approximately twice as high fraction as among the total number loci of W/1+) had significant different mean body lengths between genotypes. Among these, 30 (88.2%) had significantly higher frequency of the largest genotype in the W/1+ sample than in W/0+ (Tables 1 and S4). Just 10 candidate loci (6.7% of candidates from the W/0+ vs. W/1+ comparison) had significant genotypic body length differences in the W/0+ sample and similarly, 19 in the H/1+ sample, that is, 6.1% of the candidate loci pooled from the two pairwise analysis including H/1. In addition, two candidate loci from the hierarchical analysis had genotypic body length differences in the H/1+ sample.

Strikingly, all candidate loci from the W/0+ vs. W/1+ comparisons were nonsignificant after SGoF correction, and one candidate locus only was detected based on the BAYESIAN analysis, differing from the other sets.

Three loci showed corresponding body length differences between genotypes in the W/1+ and the H/1+ sample, and these were loci no 675 (AG larger than AA, *p *=* *.012–.030), 962 (AA larger than GG, *p *=* *.006–.049), and no 3492 (AG larger than AA, *p *=* *.003–.047). The latter two had higher frequency of the largest genotype in W/1+ than in W/0+. In one candidate locus (no 2497), the length difference was opposite in W/0+ and W/1+, and in one candidate locus (no 2841) the length difference was opposite in W/1+ and H/1+.

Under a hierarchical model, 203 (6.6%) SNP loci were detected as candidates for selection, and 26 (0.9%) of the tests were significant after SGoF correction. Under a finite island model, 231 (7.6%) outliers were significant, and, 45 (1.5%) tests were significant after SGoF correction, and 20 of 26 candidates of the hierarchic model analysis were included among the candidates under the finite island model (Figure 2). BAYESCAN analysis detected just one (W/0+ vs. W/1+) to five (W/1+ vs. H/1+), totally 10, candidates of selection (demanding log10(BF) >0.5), and all of them were included among the candidate loci detected in ARLEQUIN after SGoF correction, except the one detected by BAYESCAN in the W/0+ vs. W/1+ set (Table S2). The overlap between the SGoF corrected sets of the ARLEQUIN analysis (Table 2, with locus number referring to names in Table S1) shows that the largest overlap included the results of the hierarchical analysis. Only two of these loci had significant genotypic length differences in the W/1+ sample, and one (no 3526) had higher frequency of the largest genotype in W/1+ compared with W/0+. Strikingly, no significant outliers indicated balancing selection.

**Table 2:** Numbers of the SGoF corrected candidate loci (*S* = 16) overlapping between different sets of outlier analysis. The number of loci with genotypic length difference are boldfaced

Groups of loci	Locus number
(W/0+ vs. H/1+) ∩ (W/0+ vs. H/1+)	925, 2022, 3024, 3631, 3644
Hierarch ∩ (W/0+ vs. H/1+)	149, 688, 826, 925, 1354, 1544, 1830, 1882, 2017, 2022, 2256, 3631, 3644
Hierarch ∩ (W/1+ vs. H/1+)	925, 1370, 1415, 1828, 1862, 2022, 3320, **3526**,** 3613,** 3631, 3644
Hierarch ∩ (W/0+ vs. H/1+) ∩ (W/1+ vs. H/1+)	925, 2022, 3631, 3644

### 3.2 Genetic structure

Genetic differentiation expressed as global *F*_ST_ was 0.024 (*p *<* *.001), and the pairwise *F*_ST_ ‐ values between sample groups were all significant, and they were higher when based on selection candidate loci than when based on noncandidate loci (Table 3).

**Table 3:** Pairwise differentiation as *F*_ST_ between 0+ (W/0+), 1+ (W/1+) and hatchery‐reared (H/1+) brown trout based on two different groups of SNP markers, loci detected as candidates of selection, and loci assumed to be neutral (Noncandidates)

*F*_ST_	Analyzed	W/0+	W/1+
W/1+	All loci	0.0048	
	Candidate loci	0.0291	
	Noncandidates	0.0036	
H/1+	All	0.0334	0.0312
	Candidate loci	0.1145	0.1125
	Noncandidates	0.0226	0.0235

**Figure 4:**
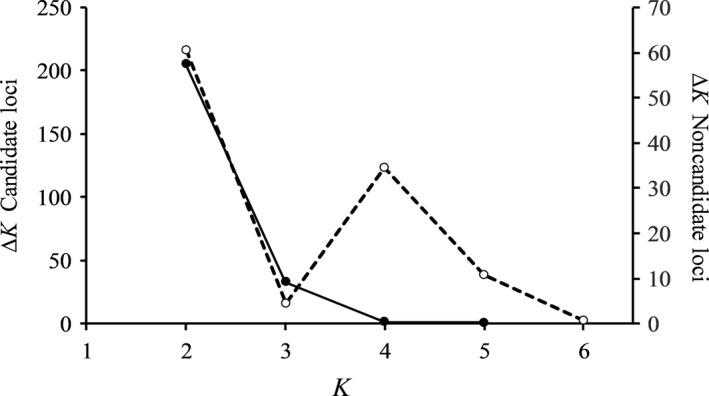
Determination of the number of clusters based on Δ*K* from STRUCTURE analysis based on 442 loci detected as candidates of selection (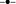) and based on 3429 loci assumed to be neutral (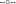)

**Figure 5:**
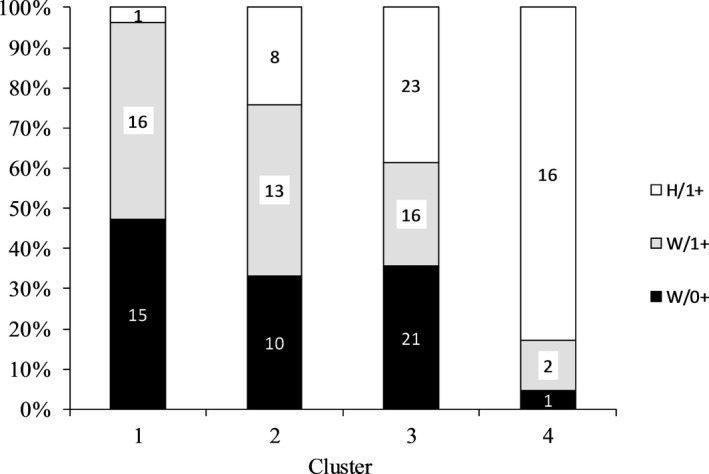
Percent of individuals of each sample group (W/0+, W/1+, and H/1+) assigned to the four SNP‐based clusters (lower panel) identified by STRUCTURE software

STRUCTURE analysis of the three sample groups suggesting the “best fit” number of clusters based on Δ*K* showed a maximum at *K *=* *2 and at *K *=* *4. When analyzing the 442 candidate loci only, Δ*K* peaked at *K *=* *2, whereas Δ*K* peaked at both *K *=* *2 and *K *=* *4 when analyzing the remaining 3,429 presumably neutral loci (Figure 4). With *K *=* *2 the Cluster 1 comprised 67.7%, 66.0% and 17.9%, respectively, of the sample groups, that is, Cluster 1 was primarily a wild fish cluster, whereas Cluster 2 was dominated by the hatchery group. One cluster was dominated by the hatchery group also with *K *=* *4 (Figure 5).

**Figure 6:**
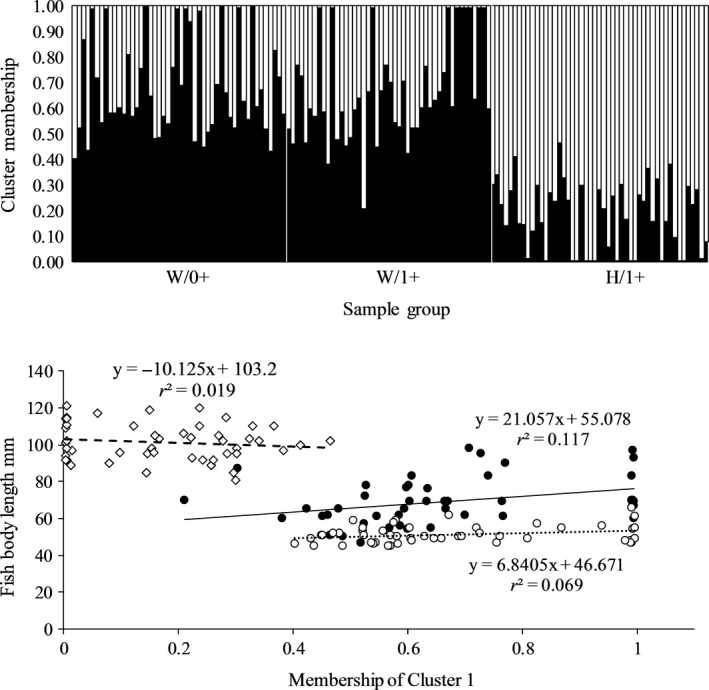
Assignment probability of individual W/0+, W/1+, and H/1+ brown trout to the two *K* = 2 clusters (upper panel), and the fish body length of W/0+ (○), W/1+ (●) and H/1+ (◇)_ specimens plotted as function of their membership to Cluster 1

**Figure 7:**
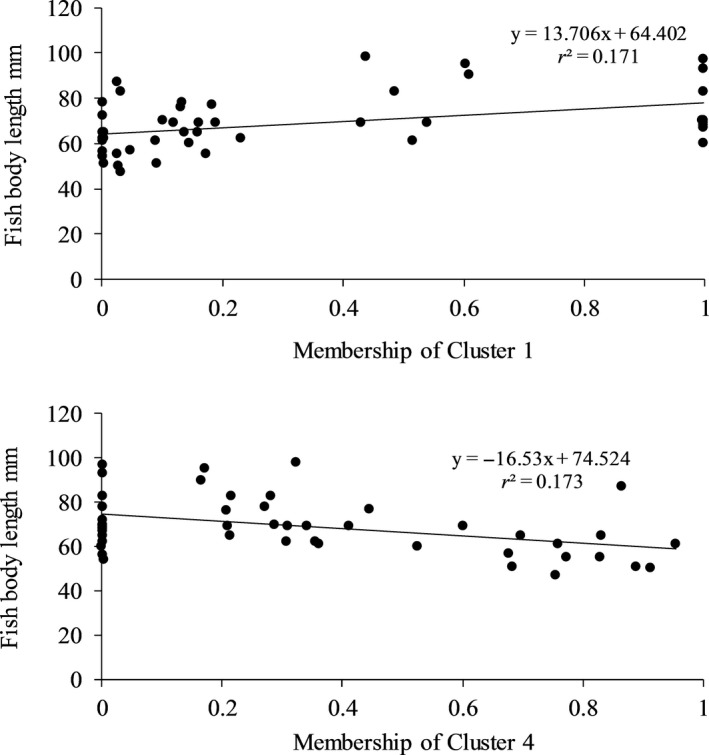
Fish body length of W/1+ specimens plotted as function of the assignment of two clusters of the *K* = 4 structure

Concerning the *K *=* *2 structure revealed from the candidate loci, fish body length of the W/1+ group correlated positively with estimated membership of one cluster (*F*_1,45_ **=* *6.0, *p *<* *.05, Figure 6), and consequently negatively with the other cluster membership. With the *K *=* *4 structure, fish length of W/1+ specimens was positively correlated (*F*_1,45_ **=* *9.28, *p *<* *.01) with the membership of Cluster 1 (with the lowest representation of H/1+, Figure 6), and negatively (*F*_1,45_ **=* *9.38, *p *<* *.01) with the membership of Cluster 4 (with the highest representation of H/1+). The coefficients of variation indicate that the memberships of the two clusters of the *K *=* *4 structure explain slightly more of the variation (17.1%–17.3%, Figure 7) than the membership of the *K *=* *2 cluster (11.7%; Figure 6).

BOTTLENECK software analyzes yielded results demonstrating significant excess of heterozygotes compared with the prediction of all three models (*p *<* *.001) indicating recent bottleneck events in all sample groups.

## 4 DISCUSSION

Wild brown trout caught in the stream Sagbekken in June (W/1+) were on average 19.4 mm larger than their relatives of the same cohort (W/0+) caught eight and a half month earlier (mostly winter conditions) in the same habitat. It is interesting to compare the two samples, in an attempt to reveal what happened with the wild brown trout during its first winter in the stream habitat. Survival during first winter has been shown to be size selective in salmonids (Hunt, 1969; Johnston, Bergeron, & Dodson, 2005; Meyer & Griffith, 1997), although size‐selective mortality may be masked by high nonselective mortality (Johnston et al., 2005; Søgard, 1997). Also, comparing offspring from the 24 wild brown trout, after random mating and breeding in a hatchery are likely to provide insight into the effect of relaxed selection. The analysis of 3,871 SNP loci makes it possible to relate body size to genotypes and to detect loci as candidates of selection, which in this study was based on outlier *F*_ST_ by means of ARLEQUIN and BAYESCAN software.

Among the 34 (22.7%) candidate loci from the W/0+ vs. W/1+ set with genotypic length differences in the W/1+ sample, almost all (88.2%) had higher frequency of the largest genotype in the W/1+ sample than in W/0+. Further, the fraction of loci with genotypic length was more than three times as high in the W/1+ sample compared with W/0+ where 6.7% of the candidate loci showed genotypic length differences. It was also more than twice as high as in the H/1+ sample where genotypic length differences were detected in 7.4% of the candidate loci (of the group of 271 candidate loci when excluding the overlapping with W/0+ vs. W/1+). The increased frequency of the largest wild‐caught genotypes from the autumn (W/0+) to the spring (W/1+) sample suggests that the wild‐grown genepool was subject to selection due to size‐selective mortality between the sampling occasions.

The weaker relationship between genotype and length in the W/0+ sample compared with W/1+ may in part be due size‐selective sampling by means of electrofishing, under‐sampling smaller individuals in the cohort (Bohlin et al., 1989). Nevertheless, none of the genotypic size differences in candidate loci of W/0+ corresponded to differences in W/1+. There were genotypic size differences of a higher number of candidate loci in the H/1+ sample, but of these 20 loci, only two showed body length differences corresponding to that in W/1+. It may be concluded that the genotypic‐specific expressions of body length varied substantially between the two environmental conditions. There were three exceptions though, where the same genotypes being largest in both W/1+ and H/1+, and these loci (no 675, 962 and 3,492) may potentially be linked to growth capacity independent of environmental factors.

The indication of selection based on outlier *F*_ST_ was generally weaker in the W/0+ vs. W/1+ pair than in the other pairwise analysis, expressed by the fact that only one locus was detected by means of the conservative BAYESCAN method, and no outlier *F*_ST_ was significant after to the SGoF correction. The increased frequency of the largest genotype in W/1+ compared with the W/0+ sample, nevertheless suggests an effect of selection, which is notable, and it seems like the conservative statistics in this case lead to statistical Type II error (i.e. accepting H_0_ when it is false). All significant outlier *F*_ST_ estimates revealed by means of ARLEQUIN and SGoF+ corrected (Narum & Hess, 2011), therefore should be included when observable traits are considered.

The genetic differentiation, expressed as *F*_ST_, was significant between all sample pairs, and it was higher when based on candidate loci than when based on noncandidates, not surprisingly, as candidate detection was based on (outlier) *F*_ST_. The differentiation was largest between the wild fish groups (W/0+ and W/1+) and the hatchery fish (H/1+). This differentiation was emphasized by the Bayesian structure, with a cluster dominated by hatchery fish, both with *K *=* *2 and *K *=* *4 structure. The *K *=* *2 structure based on candidate loci indicate, and the differentiating process acted more strongly in the hatchery group than on the stream living specimens. This corresponds to a previous survey, where SSR‐based differentiation between hatchery fish and wild brown trout from the two nursery streams of the hatchery fish parents, Sagbekken and Mogardsbekken, were *F*_ST_ **=* *0.052 and 0.063. This was more than four times the *F*_ST_ **=* *0.013 between the wild brown trout from Sagbekken and Mogardsbekken (Linløkken & Johansen, 2010). The outlier *F*_ST_, however, quantifies genetic differentiation between groups, but provides little information about the causal mechanisms imposing differentiation. The hatchery fish were bred from an effective number of breeders that was approximately half of that of the wild fish (Linløkken et al., 2016), with potential effect on genetic drift. Further, differentiation may result from the absence of sexual selection and/or differentiating selection mechanisms imposed under artificial spawning compared to what occurs under natural spawning. The genepool of the resulting offspring therefor may be very different from what results from natural spawning (Araki et al., 2008; Lamaze, Garant, & Bernatchez, 2013; Wedekind, Rudolfsen, Jacob, Urbach, & Muller, 2007). With a mortality of <5% in the hatchery, there was hardly any postfertilization selection affecting the H/1+ group, that is, phenotypic misfits in the wild, could survive well in the hatchery.

The significant correlation between W/1+ body length and the individual memberships of two clusters, with both *K *=* *2 and 4 structure, also suggested that some SNP loci were linked to growth capacity and were expressed differentially between wild and hatchery‐reared brown trout. This finding agrees with other studies demonstrating highly differentiated selection regimes in salmonid hatcheries compared with the wild (Besnier et al., 2015; Sundström, Petersson, Höjesjö, Johnsson, & Järvi, 2004), with possible negative long‐term introgression consequences for wild populations exposed to repeated stocking of hatchery‐reared individuals (Araki et al., 2008; Lamaze et al., 2013; Wedekind et al., 2007). In nature, adaptation to actual temperature regime is crucial (Bærum et al., 2016; Jensen et al., 2008; Koskinen, Haugen, & Primmer, 2002). The lack of natural selection under hatchery conditions may also lead to survival of maladapted behavior types that normally would not survive in nature. For instance bold behavior types may be beneficial in a hatchery environment as food is not limited and predation risk nonexistent (Sundström et al., 2004). Individuals with such risk‐prone behavior are probably likely to be subject to predation in the wild.

Our results suggest that winter and spring conditions in the rearing stream Sagbekken favor genotypes coding for expressions of phenotypic values of a combination of physiological and behavioral traits (possibly linked to feeding activity) at low temperatures, and through this affects the mean body size of the cohort. This differs from the even larger hatchery fish, in which associations between size and genotype were found mostly at other candidate loci than in the W/1+ sample. Survival is commonly positively related to the weight of fish in early stages (Lorenzen, 1996), and higher mortality rates during the first winter and spring of slow‐growing individuals may explain the genotype frequency differences between the W/0+ and W/1+. The relationship between genotypes and body length was detected in several loci in this study, although for the majority of candidate loci it was not so. The loci detected in several pairwise, and in the hierarchical ARLEQUIN analysis, after correction, and in the BAYESIAN analysis as well, are most probably linked to traits of importance to individual fitness.

Based on the results of a large‐scale analysis of these three populations, further analysis should be conducted on selected SNPs, depending on the purpose of the study. SNPs under selection, possibly linked to some observable traits, can be used to monitor the effects of environmental changes (including human‐induced habitat alterations) and the introduction of pathogens as well as natural selection. The comparability of SNPs across laboratories (Morin, Martien, & Taylor, 2009) makes it easy to compare studies on traits linked to genes from different regions worldwide.

## CONFLICT OF INTEREST

None declared.
